# Comparative analysis of intraoperative fluoroscopic vs. Anatomical landmark positioning methods in MPFL reconstruction for recurrent patellar dislocation

**DOI:** 10.1186/s12891-025-09156-z

**Published:** 2025-09-30

**Authors:** Ruke Lin, Ping Fu, Xinfu Zhang, Yajie Wu, Xibei Lin, Daohong Zhao

**Affiliations:** Department of Orthopaedics, The Second Affiliated Hospital of Kunming Medical University, Kunming, 650032 China

**Keywords:** MPF, Recurrent patellar dislocation, Schöttle point, Fluoroscopy, Anatomic landmark, Reconstruction, 3D-CT

## Abstract

**Objective:**

A retrospective analysis was conducted to evaluate the application of the intraoperative fluoroscopic positioning and anatomical landmark positioning methods in medial patellofemoral ligament (MPFL) reconstruction for recurrent patellar dislocation. The aim was to summarize the positioning accuracy and clinical efficacy of each method, to serve as a reference for femoral positioning.

**Method:**

We conducted a retrospective analysis of a cohort comprising 75 patients who underwent treatment for recurrent patellar dislocation at our institution between January 2014 and September 2020.Based on the different positioning methodologies utilized for identifying the MPFL femoral footprint, the included patients were systematically allocated to either the fluoroscopy group or the palpation group.Preoperative evaluations and assessments at the latest follow-up encompassed the International Knee Documentation Committee (IKDC) score, Lysholm score, and Kujala score for both groups.We utilized immediate postoperative CT scans for our evaluations. A total of 48 knee 3D-CT scans were acquired using Mimics Medical 21.0 for both groups. From these scans, we constructed a standard lateral Schottle point on a 3D-CT image. To assess the relative positions between the actual and standard location points in both groups, we established a coordinate system based on a simplified, constructed standard point baseline (as illustrated in Chart e). Subsequently, the relative positions of the actual points were evaluated.

**Result:**

All 75 patients were followed up for a period ranging from 36 to 96 months( mean: 62.27 ± 21.36 months). Significant improvements were observed in the IKDC score, Lysholm score, and Kujala score from preoperative to the latest follow-up (*p* < 0.05) (Table 2), indicating statistical significance.Furthermore, the latest follow-up revealed no significant differences in knee function scores between the two groups (*P* > 0.05) (Table 3). Similarly, the latest evaluation showed no significant differences in knee function scores between patients undergoing MPFLR and MPFLR + TTO In their respective groups (*P* > 0.05) (Table 4).CT-3D reconstruction was conducted on 48 postoperative patients (24 in the fluoroscopy group and 24 in the palpation group). Evaluation of the positioning revealed that most cases in the palpation group were located in quadrants 1 and 3, whereas those in the fluoroscopy group were primarily distributed across quadrants 1, 3, and 4 (*p* < 0.05), indicating statistical significance.In the palpation group, the isometric distance was 3.90 ± 2.17 mm, with an isometric rate of 75%. In the fluoroscopy group, the isometric distance was 7.55 ± 3.94 mm, with an isometric rate of 29.2%.The femoral tunnel isometric rate was significantly higher in the palpation group, at 75%, compared to 29.2% in the fluoroscopy group. among the two positioning methods, there was no statistical difference in the positioning of the femoral footprint at the anterior and posterior ends of the standard point, but there was a statistical difference at the proximal and distal ends (*P* < 0.05).

**Conclusion:**

Clinical outcomes significantly improved and were similar in both groups. Nevertheless, the palpation of femoral anatomical landmarks exhibited superior convenience and efficiency for experienced sports medicine practitioners, and additionally, it frequently achieved a more isometric femoral footprint than fluoroscopic positioning in certain scenarios.

## Introduction

Recurrent patellar dislocation(RPD) is a common condition in sports medicine, frequently affecting teenagers, especially female patients [1]. It is the primary cause of knee pain among juveniles [2]. Recent studies have underscored the pivotal role of the medial patellofemoral ligament (MPFL) in preventing lateral patellar dislocation [3]. The MPFL accounts for 50–60% of the restraint limits on external patellar migration [4]. Similar findings were reported by Desio et al., who concluded that the MPFL provides a 60% limiting force [5]. Currently available evidence indicates that approximately 95% of patients sustain an injury to the MPFL following their first episode of patellar dislocation [6]. Furthermore, 71% of these patients sustain cartilage damage [7], which renders them susceptible to subsequent dislocations following an initial incident. MPFL reconstruction is currently regarded as the principal surgical intervention for treating RPD. The anatomical reconstruction of the MPFL plays a vital role in maintaining medial soft tissue stability and ensuring successful surgical outcomes. As a result, MPFL reconstruction has emerged as the primary procedure for treating RPD, owing to its favorable postoperative clinical effects and low risk of redislocation [8].

There are various techniques available for MPFL reconstruction. However, regardless of the chosen method, precise positioning of the patella and femoral footprint during reconstruction is crucial to ensure anatomical restoration. Achieving isometry, particularly with respect to length, in ligament reconstruction significantly enhances the stability of the reconstructed patellofemoral joint [9]. A study has demonstrated that accurate positioning of the femoral footprint in MPFL reconstruction plays a pivotal role in achieving isometricity and, consequently, ensures stability within the patellofemoral joint [10].

There are two primary positioning methods for femoral footprint in MPFL reconstruction: the first method involves intraoperative fluoroscopic positioning, commonly utilized at Schöttle [11] points (Fig. 2.chart 1). These points are defined as the intersection of 1 mm anterior to the extension of the posterior cortex, 2.5 mm distal to the contact point of the medial condyle and the posterior cortex, and 5 mm proximal to the most posterior point of the Blumensaat line. However, a study found that locating Schottle points using fluoroscopy and obtaining standardized lateral femoral images during surgery can be challenging [12]. Even slight rotational deviations in lateral fluoroscopy can lead to significant positioning errors deviating from the standard point. The second method is palpation positioning based on anatomical landmarks [13] (Fig. 2.chart 2), Which depends on establishing positional relationships among the adductor tubercle(AT), the femoral medial epicondyle(FME), and the attachment site of the MPFL on the femur. Many scholars such as Nomura [14] and Panagiotopoulos [15] have suggested that normal MPFL insertion occurs between the femoral medial epicondyle(FME) and the adductor tubercle.

This study aims to evaluate the clinical efficacy and positioning accuracy of two femoral positioning methods — intraoperative fluoroscopic and anatomical landmark-based techniques — in MPFL reconstruction to determine which method offers superior outcomes in recurrent patellar dislocation.

## Information and methods

### General data and methods

We retrospectively analyzed 75 patients (27 males, 48 females; female-to-male ratio ≈ 1:1.77) with RPD at our hospital from January 2014 to September 2020. Their ages ranged from 15 to 45 years, with a median of 19 (Table 1).

**Table 1:** General patient data

Group	Gender		Age, year	Patella Wiberg type			Trochlea Morphology Dejour type				TT-TG distance/mm	Surgical technique	
	M	F		I	II	III	A	B	C	D		M	M + T
Flu-group	15	25	21.63 ± 7.50	5	28	7	12	17	8	3	19.8 ± 4.70	30	10
palpation group	11	24	21.46 ± 6.07	2	19	14	19	8	7	1	18.5 ± 4.60	21	14
	χ^2^ = 0.304		t=−0.109	χ^2^ = 1.819			χ^2^ = 2.015				t = 1.192	χ^2^ = 1.93	
P	0.582		0.914	0.162			0.136				0.237	0.165	

M: MPFLR M + T: MPFLR + TTO *MPFLR* Medial patellofemoral ligament reconstruction

*TTO* Tibial tubercle osteotomy

*Flu- *fluoroscopy, *M* male, *F* female

Inclusion criteria: A clinical diagnosis of recurrent patellar dislocation included two or more dislocations requiring reconstruction of the medial patellofemoral ligament.

Exclusion criteria: (1) history of previous knee surgery; (2) revision surgery; (3) immature epiphysis; (4) tumor, infection, or rheumatoid arthritis; (5) habitual patellar dislocation; (6) patellar fracture.

**Fig. 1:**
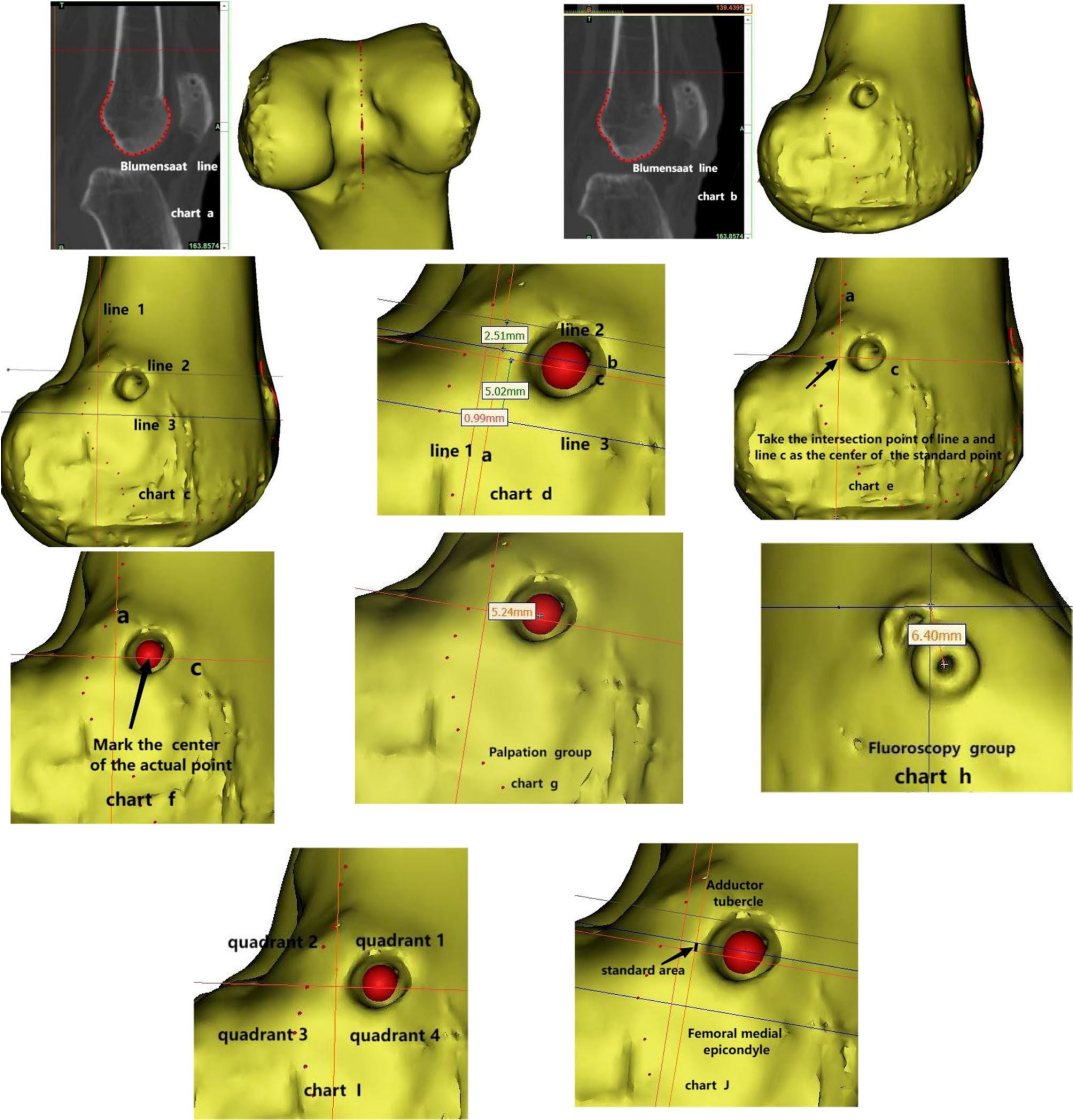
Chart ab: The Blumensaat line is projected into the 3D reconstruction.Chart c:Line 1 represents the extension of the posterior cortex. Line 2 and Line 3 are perpendicular to Line 1. Line 2 marks the contact point between the medial condyle and the posterior cortex, while Line 3 corresponds to the most posterior point of the Blumensaat line.Chart d: Line a, anteriorly aligned and parallel to Line 1, is positioned at a consistent distance of 0.99 mm; Line b, distally oriented and parallel to Line 2, maintains a separation of 2.51 mm; Line c, proximally aligned and parallel to Line 3, is situated at an interval of 5.02 mm. These three lines—Line a, Line b, and Line c—collectively demarcate the isometric region of interest within the anatomical assessment.Chart e:The intersection point of Line a and Line c is considered the center of the standard point. Chart f:Identify and mark the central point of the actual point.Chart g:In the palpation group, the distance between the standard point and the actual point is 5.24 mm.Chart h:In the fluoroscopy group, the distance between the standard point and the actual point measures 6.40 mm. Chart I:Schematic illustration of quadrant differentiation.Chart J:Graphical depiction of the standard area

Methods: After obtaining consent from the hospital ethics committee, we utilized the hospital’s HIS system to screen cases based on the previously proposed inclusion and exclusion criteria. Seventy-five eligible patients were selected, and the immediate postoperative CT images were collected based on different positioning methodologies.A total of 48 knee CTs were reconstructed by a single observer using Mimics Medical 21.0 software, and the standard lateral Schöttle point was constructed in the 3D-CT scans(Fig. 1 Chart a, b,c, d,e and f).We measured the distance between the actual point center and the standard point, measured each value three times, and then took the average (Fig. 1 Chart g and h); an isometric point was defined as a location exhibiting a deviation of no more than 5 mm from the reference standard [13]. The coordinate system was established using a simplified baseline derived from the Schöttle point. Quadrant 1 represented the proximal-anterior position, quadrant 2 represented the proximal-posterior position, quadrant 3 represented the distal-posterior position, and quadrant 4 represented the distal-anterior position; quadrants 1 and 2 were proximal, while quadrants 3 and 4 were distal; quadrants 1 and 4 were anterior, whereas quadrants 2 and 3 were posterior (Fig. 1, Chart I). The relative positions of the actual points in both groups were evaluated (Table 5).

### Surgical methods

All procedures were performed by the same experienced sports medicine physician.

#### Palpation group

**Fig. 2:**
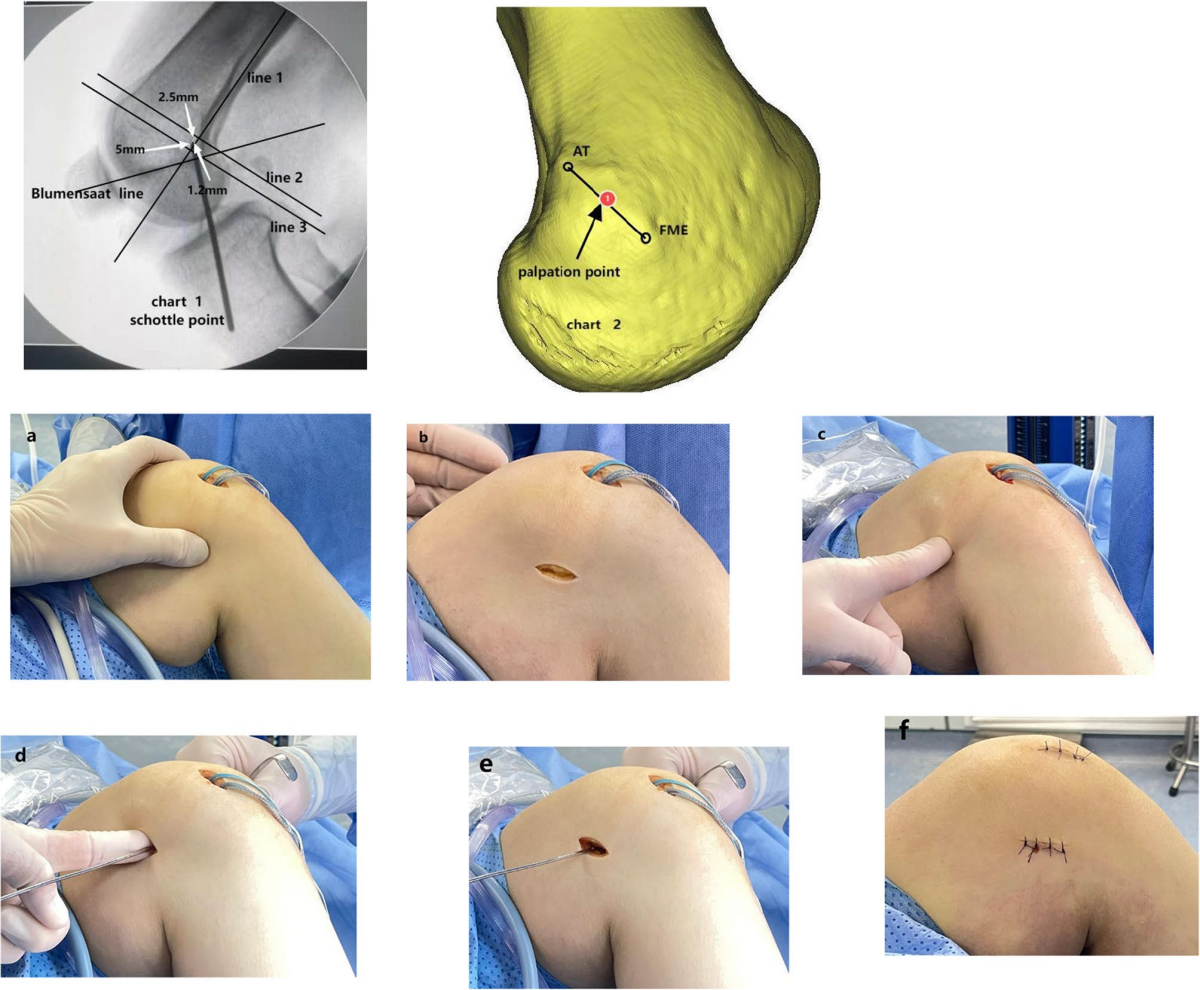
Chart 1: The fluoroscopy method is demonstrated as follows: The Schottle point is the intersection that is 1.2 mm anterior to line 1, 2.5 mm distal to line 2, and 5 mm proximal to line 3. Chart 2 :Demonstration of the palpation method: The palpation point (①) is the midpoint between the femoral medial epicondyle(FME) and the adductor tubercle(AT), and it is parallel to the long axis of the femur. **a** Palpation of the FME; **b** A 2 - cm longitudinal incision is made proximal to the FME; **c** The FME, AT, and saddle area are identified with the fingers; **d** A guide pin is inserted into the midpoint of the FME and the AT according to palpation; **e** The femoral tunnel placement is identified. **f** An incision approximately 2–3 cm in length is made postoperatively. FME: femoral medial epicondyle AT: adductor tubercle

Initially, arthroscopy was employed to evaluate cartilage damage, the presence of loose bodies (corpora liberum), soft tissue injuries, and the congruency of the patellofemoral articulation. A 3-cm longitudinal incision was made inferior to the tibial tubercle. Subsequently, the semitendinosus tendon was meticulously harvested, excised, and refined to create the autologous graft.If The preoperative TT-TG distance exceeded 20 mm, and thus tibial tubercle medialization was performed. Most patients did not require adjustment of patellar height. Three patients had a Caton index > 1.2, so we empirically distalized the tibial tubercle by approximately 0.3–0.5 cm simultaneously.A 2 - cm longitudinal incision was made along the medial edge of the patella. Two polyether ether ketone (PEEK) belt rivets were inserted into the patella at the middle and upper third of the medial border, and then the middle portion of the autograft was fixed by the rivet tail lines.Following the palpation of the FME, a 2 - cm longitudinal incision was created proximal to it; Through this incision, the FME was identified with the fingers again, and then the AT was palpated along the long axis of the femur, while carefully palpating the saddle area [16] between the two.A guide pin was inserted into the Midpoint between the FME and the AT as the femoral tunnel placement(Fig. 2 chart 2). A 6-mm reaming was performed in the femoral tunnel. Blunt dissection meticulously created a soft tissue tunnel from the patellar footprint to the femoral tunnel, maintaining joint capsule integrity and guided by needle fixation for graft insertion. Upon arthroscopic verification of the optimal patellar trajectory and patellofemoral articulation congruency, the graft was implanted into the femoral tunnel and fixed utilizing a 6 mm bioabsorbable interference screw under 90 degrees of knee flexion (Fig. 2a, b,c, d,e, f.)

#### Fluoroscopy group

The tibial tubercle osteotomy and patellar procedures were congruent with those employed in the palpation group. The femoral tunnel placement was determined by the Schöttle method [11]. A longitudinal incision was made around the FME. Standardized lateral radiographs were taken using C-arm fluoroscopy, with both posterior condyles projected in the same plane.A guide pin was positioned in the radiograph allowing correction for position.The Schöttle point was identified on the radiograph. Utilizing C-arm fluoroscopic guidance, the guide pin underwent iterative adjustment to ensure precise positioning of the femoral tunnel at the designated Schöttle point (Fig. 2 Chart 1). Subsequent operations were carried out in the same manner as those in the touch group.

### Postoperative management

Postoperatively, a knee orthosis was applied, and quadriceps femoris contraction exercises were instigated upon emergence from general anesthesia. Two days following the surgical procedure, patients commenced partial weight - bearing activities upon ambulation. Knee flexion exercises were initiated 2–3 days post - operation, with the goal of attaining 90 degrees of knee flexion within a three - week period. Four weeks after surgery, all patients presented to the hospital for a follow - up visit to receive guidance on knee stability control, muscle strength rehabilitation, and normal gait exercise training. Three months following the operation, a simple physical exercise protocol was implemented.

### Observing indicators

Comparing the isometry rates of the femoral tunnel in both groups and the central tendency in the positioning quadrants. Comparing the preoperative and latest follow - up Lysholm score, IKDC score, and Kujala score of the knee joint. The distance between the standardized point on the femur and the actual femoral point, referred to as the isometric point distance, was measured on the postoperative CT 3D reconstruction images. If the isometric point distance exceeded 5 mm, it was deemed a non - isometric tunnel; if the isometric point distance was less than 5 mm, it was considered an isometric tunnel [13]. A smaller isometric point distance signified more accurate positioning of the femoral tunnel.

## Results

### Group results

A total of 75 patients were enrolled: 40 patients in the fluoroscopy group and 35 in the palpation group. Among them, immediate postoperative knee CT scans were collected from 48 patients, with 24 patients in each of the fluoroscopy and touch groups.

### Description of the statistical methods

Data analysis was conducted using SPSSAU. Normally distributed quantitative data were described as the mean ± standard deviation. Independent - samples t - tests were used for comparisons between groups, and paired t - tests were employed for comparisons between preoperative and final follow - up data. Count data were described in terms of the number of cases and rates, and group data were compared using the chi - square test or Fisher’s exact test. A two - sided *P* < 0.05 was regarded as statistically significant. Improvement in the Kujala score was considered the primary outcome [17]. Sample size calculation was performed according to previous studies based on a significant difference in the mean Kujala score (8 ± 8 scores) [18, 19]. At least 17 cases in each group were required to detect significant changes with 80% power and 95% confidence.

### Efficacy and safety evaluation results

The study subjects exhibited no nonunion of the tibial tubercle, no lower - limb deep vein thrombosis, no joint infection, and no joint stiffness, and were capable of returning to simple exercise 3 months after surgery.The average postoperative follow - up duration was 62.27 months. Postoperatively, CT 3D reconstructions were acquired in 48 cases (24 cases in the fluoroscopy group and 24 cases in the palpation group), and the positioning of both groups was assessed. The palpation group was predominantly in quadrant 1 and quadrant 3, whereas the fluoroscopy group was mainly in quadrant 1, quadrant 3, and quadrant 4 (*P* < 0.05), which indicated a statistically significant difference.In the palpation group, the isometric distance was 3.90 ± 2.17 mm, with an isometric rate of 75%. In the fluoroscopy group, the isometric distance was 7.55 ± 3.94 mm, with an isometric rate of 29.2%. The proportion of isometric tunnels was higher in the palpation group than in the fluoroscopy group. among the two positioning methods, there was no statistical difference in the positioning of the femoral footprint at the anterior and posterior ends of the standard point, but there was a statistical difference at the proximal and distal ends (*P* < 0.05) (Table 5). Significant improvements were observed in the IKDC score, Lysholm score, and Kujala score from preoperative to the latest follow-up (*p* < 0.05) (Table 2), indicating statistical significance.Furthermore, the latest follow-up revealed no significant differences in knee function scores between the two groups (*P* > 0.05) (Table 3). Similarly, the latest evaluation showed no significant differences in knee function scores between patients undergoing MPFLR and MPFLR + TTO In their respective groups (*P* > 0.05) (Table 4).

**Table 2:** Compare the clinical outcomes preoperatively and postoperatively (at the latest follow - up) in the two groups respectively

Group	IKDC score		Lysholm score		Kujala score	
	Fluoroscopy group	palpation group	Fluoroscopy group	palpation group	Fluoroscopy group	palpation group
Pre-	50.25 ± 13.20	53.06 ± 15.17	51.20 ± 19.32	51.63 ± 20.05	43.17 ± 26.13	48.37 ± 24.52
Post-	71.80 ± 3.99	73.97 ± 4.03	91.08 ± 6.45	92.23 ± 6.54	92.75 ± 5.54	94.00 ± 5.04
T-value	−13.79	−9.90	−18.709	16.807	−14.868	−13.353
P-value	0.000	0.000	0.000	0.000	0,000	0,000

*Pre- *preoperatively, *post- *postoperatively

**Table 3:** Compare the postoperative (at the latest follow-up) clinical outcomes in the two groups

Group	Case	Follow-up	IKDC score	Lysholm score	Kujala score
			Postoperative	Postoperative	Postoperative
Flu-group	40	62.33 ± 20.26	71.80 ± 3.99	91.08 ± 6.45	92.75 ± 5.54
palpation group	35	62.16 ± 21.41	73.97 ± 4.03	92.23 ± 6.54	94.00 ± 5.04
T-value	/	0.066	1.773	0.417	0.715
P-value	/	0.947	0.085	0.679	0.479

*Flu-* fluoroscopy

**Table 4:** Compare the postoperative (at the latest follow-up) clinical outcomes of patients who underwent MPFLR and MPFLR + TTO in the two groups

Group	IKDC score		Lysholm score		Kujala score	
	Fluoroscopy group	Palpation group	Fluoroscopy group	Palpation group	Fluoroscopy group	Palpation group
M	72.00 ± 3.86	74.79 ± 3.66	91.10 ± 6.76	94.50 ± 6.60	92.00 ± 5.81	95.57 ± 4.72
M + T	72.00 ± 5.27	74.86 ± 4.00	91.60 ± 7.62	92.50 ± 6.43	92.60 ± 6.47	95.00 ± 4.56
T-value	0.000	−0.074	−0.152	0.916	−0.222	0.376
P-value	1.000	0.942	0.883	0.376	0.829	0.713

M: MPFLR M + T: MPFLR + TTO, *MPFLR* Medial patellofemoral ligament reconstruction

*TTO* Tibial tubercle osteotomy

## Discussion

In this study, the Schöttle point was constructed via three - dimensional CT to compare the isometric distance and rate between intraoperative fluoroscopy and anatomical landmark positioning methods as well as the relative spatial positions of actual points, which differed slightly from the traditional view. Experienced sports medicine doctors could also obtain high isometric rates by palpating anatomical landmarks, and there was no significant difference in knee function scores at the latest follow - up between the two methods. Meanwhile, this study showed that sports medicine doctors could plan the position and design MPFL reconstruction surgery through preoperative 3D CT to improve effectiveness (Table 5).

**Table 5:** Compare the postoperative position and quadrants

Group	Gender		Distance to standard points/mm	Position				Quadrants				Age, year
	M	F		anterior	posterior	Proximal	distal	1	2	3	4	
Flu-group	8	16	7.55 ± 3.94	16	8	9	15	7	2	6	9	20.75 ± 7.43
palpation group	6	18	3.90 ± 2.17	14	10	15	9	12	3	7	2	20.25 ± 5.86
	χ^2^ = 1.000		t=−3.597	χ^2^ = 1.371		χ^2^ = 5.531		χ^2^ = 21.379				t=−0.311
P	0.317		0.002	0.242		0.019		0.011				0.758

*Flu-* fluoroscopy, *M* Male, *F* Female

At present, anatomical reconstruction was a primary approach for MPFL reconstruction.During this process, the tunnel placement at both ends of the patellofemoral joint determined the normal shape, direction, initial length, and isolength of the graft. Therefore, whether the theoretical isometry between the actual and standard positioning points directly affected the isolength of MPFL reconstruction was crucial [20]. The isolength, the reconstructed ligament, is basically unchanging in knee movement. Currently, no definitive definition of the graft’s isolength in MPFL reconstruction exists. Ellera et al. [21] proposed that the length variation of the MPFL during knee activity should be less than 5 mm. Some studies have reported that the mean length change of the native MPFL of the knee from 0 - degree flexion to 110 - degree flexion was 1.5–2 mm, so a change of less than 2 mm was regarded as isometric motion [13]. Some scholars [22–24] held that there were anatomical variations, and the isolength requirement for MPFL reconstruction was not very stringent; however, non-isolength and non- anatomical reconstructions could lead to numerous problems [25]such as restricted patellar mobility, loss of the knee’s dynamic range, medial patellofemoral joint hypertension, graft failure resulting in knee pain and even recurrent dislocation. Regarding isolength studies, the majority of the literature indicated that the femoral footprint had a considerable impact on the isolength of the graft, while the patellar footprint also had a certain (not insignificant as previously stated) effect on its isolength [26, 27]. Steensen et al. [28] discovered that the upper margin of the anatomical footprint of the MPFL was equivalent to the position of the upper angle of the patella, while the lower margin of the anatomical footprint was approximately equivalent to the midpoint of the inner margin of the patella, and its width was approximately 20–30 mm. Consequently, MPFL reconstruction was closer to the anatomy. Some scholars [28, 29] selected the middle and upper one - third positions of the inner edge of the patella as the double bundle tunnel placement. Inaccurate femoral footprint positioning often leads to MPFL reconstruction failure. Accurate positioning is crucial as it determines the graft’s length and isolength, knee ligament tension at various flexion degrees, and patella stability.Stephen et al. [30] found that a 5 - mm displacement of the femoral tunnel placement from the anatomical site would induce an aberrant patellar kinematics during knee motion. Thaunat et al. [27]believed that if the femoral footprint was proximal or anterior, the graft would be relaxed when the knee was extended, tension would occur during flexion, and the clinical manifestations would be anterior knee pain and limitation of knee flexion; in contrast, if the femoral footprint was distal or posterior, the graft would be tense when the knee was extended, and flexion would show asthenia and limitation of knee extension. A study [20] showed that the most isometric locations for MPFL reconstruction were the posterior and proximal footprint of the femoral MPFL.

Currently, two primary techniques are employed for femoral footprint positioning: intraoperative fluoroscopy and palpation of anatomical landmark. For the intraoperative fluoroscopy method, the Schöttle point [11] was used commonly, the MPFL footprint was within 5 mm from the center of the Schöttle point. Stephen et al. [30] proposed that with respect to the femoral condyle, the anterior - posterior distance was considered as 100%, while for the MPFL femoral footprint, it was 60% from the anterior, 40% from the posterior, and 50% from the distal end.

The palpation of anatomical landmark positioning method, such as that employed by Chassaing et al. [31] selected the posterior part of the femoral medial supporting band and the site 1 cm behind the epicondyle near the femoral footprint as the femoral insertion site for the graft. Fujino et al. [32] selected the point that is 10 mm distal to the apex of the adductor tubercle along the long axis of the femur to determine the femoral site. Wangcheng et al. [33] reported the positioning method under arthroscopic direct vision: the adductor tubercle(AT), femoral medial epicondyle(FME), and posterior edge of the femur were selected as the main anatomical landmarks, and the relative position of the MPFL femoral attachment point to these three anatomical points was directly observed under arthroscopy.Song Yifan et al. [34] located the Schöttle point without using fluoroscopy to determine the apex of the adductor tubercle(AT), where the posterior edge was identified. The posterior edge was defined as the transition zone between the medial femoral condyle and the femoral shaft. The femoral footprint was situated 8 mm distal to the apex of the adductor tubercle(AT) and 8 mm anterior to the posterior edge. Chen et al. [16] introduced the saddle area positioning technique, which involves drawing a vertical line about 12 mm distal to the line connecting the adductor tubercle(AT) and the femoral medial epicondyle(FME). A point 6 mm posterior to this vertical line can serve as an intraoperative landmark for the femoral side of the medial patellofemoral ligament (MPFL).What is the accuracy of the two positioning methods? Some studies reported that the localization accuracy was 71.3% [35] and 56.2% [36]. Interestingly, Sanchis-alfonso et al. [37] reported that the accuracy of X - ray fluoroscopy was merely 38%. It is worth mentioning that Song Yifan et al. [34] reported that the accuracy of their study without fluoroscopy positioning of Schöttle point was as high as 95.2%. In this study, 75% of the palpation group also showed 29.2% higher than the fluoroscopy group.

Anatomical landmarks are exposed by small incisions, which can increase the accuracy of palpation.The study showed that positioning by palpating anatomical landmarks without the use of fluoroscopy was also accurate [34]. Numerous studies [13, 16] have validated that the femoral footprint of the medial patellofemoral ligament (MPFL) was situated between the femoral medial epicondyle(FME) and the adductor tubercle(AT). Intraoperative palpation of these anatomical landmarks enabled direct visualization, facilitates individualized treatment planning, and closely aligns with the patient’s specific anatomy, thereby potentially enhancing the precision of tunnel placement. X - ray fluoroscopic positioning approaches the standard point step by step through continuous fluoroscopic positioning and has high accuracy theoretically.However, if we want to obtain complete overlap of the standard lateral views of the medial and lateral femoral condyles, the actual operation is very difficult.Balcarek et al. [38] conducted a fluoroscopy study of hip rotation and found that 2.5 degrees of hip adduction, abduction, and internal - external rotation could result in significant displacement of the site towards the distal, proximal, anterior, and posterior ends, respectively. Some scholars also utilized high - resolution bone geometry to provide the MPFL path length under physiological loading conditions. The calculated optimal isometric femoral insertion point exhibited a variable anatomical distribution, indicating that the optimal location for femoral MPFL graft fixation varied among different patients [24]. In conclusion, Perhaps this can explain why the palpation of anatomical landmarks positioning method is more accurate than the fluoroscopy positioning method in this study.

Consistent with previous literature and in this study, medial femoral condyle dysplasia and torsion, increased femoral inclination, patella alta, an increased TT - TG (tibial tuberosity - trochlear groove) value, and trochlear dysplasia are important factors that affect the accuracy of fluoroscopic positioning, and the specific algorithm requires further study.However, some authors’ study [39] found that despite the presence of a TT - TG value greater than 20 mm, MPFL reconstruction alone could also yield good clinical results.In MPFL reconstruction, accurate anatomical positioning serves as the foundation for achieving favorable clinical outcomes.In the absence of high-grade patellar dysplasia, anatomically positioned femoral tunnels resulted in markedly better clinical outcomes, 83% of patients were either very satisfied or satisfied with the outcome of their surgery, and 56% were able to return to sports activities after the operation [40]. Another Study [41] implied that precise femoral positioning was crucial, as malposition could trigger a host of issues, including premature joint degradation, arthritis, enduring discomfort, and repeated patellar dislocations.

According to a previous study [20] the most isometric position of MPFL reconstruction was the posterior and proximal ends of the anatomical footprint of MPFL on the femur.Therefore, in this study, positioning in quadrant 2 was identified as the most isometric, followed by quadrants 1 and 3, with quadrant 4 being the least isometric.This study indicates that among the two positioning methods, there was no statistical difference in the positioning of the femoral footprint at the anterior and posterior ends of the standard point, but there was a statistical difference at the proximal and distal ends.(*P* < 0.05). The two positioning methods also showed statistical differences when positioned in different quadrants(*P* < 0.05) (Table 5). However, because of the limited sample size, we failed to compare the correlation between positioning in different quadrants and clinical outcomes. This study also found that by obtaining the standard lateral view through three - dimensional CT and then constructing the Schöttle point as the standard point, this standard point was actually a standard area parallel to the posterior cortex of the femur. (Fig. 1 chart J). The posterior femoral cortex was located at the posterior edge of the ridge separating the femoral medial epicondyle(FME) from the adductor tubercle(AT) and was approximately parallel to the long axis of the femur. This finding was consistent with the descriptions of various anatomical landmark positioning methods reported in previous studies [31–34]. The non - standard knee may have narrowed this standard area, which is consistent with the results of the Matsushita et al. [42] study. This might explain why the palpation group in this study had a higher isometry rate than the fluoroscopy group. There was no significant difference in the postoperative clinical outcomes between the two groups, possibly due to the existence of this standard area, which enhanced the range area of positioning. Further studies with larger samples and higher levels of evidence are required for this region.

With the application of arthroscopy, we were able to directly observe the patellofemoral joint congruence, the patellar trochlear spacing, and the friction between the graft and the femoral medial epicondyle(FME). Through this, we could indirectly assess the accuracy of the positioning.Palpating the femoral medial epicondyle(FME) and adductor tubercle (AT) through small incisions could significantly improve the accuracy of the femoral footprint. Compared with the fluoroscopy positioning, which necessitated repeated fluoroscopy to obtain standard lateral slices and constantly approached the anatomical footprint, the palpation method could effectively avoid the increased radiation dose resulting from multiple exposures, prolonged surgical duration, and heightened risk of surgical infection. However, there was a certain learning curve associated with MPFL reconstruction surgery. Beginners could plan on three - dimensional CT preoperatively and identify anatomical landmarks using X - ray fluoroscopy to ensure high - precision positioning of the MPFL femoral footprint during the learning process.

This study also has certain limitations. Firstly, the Schöttle method was employed for the fluoroscopy group, while the palpation group utilized palpation of the femoral medial epicondyle (FME) and the adductor tubercle (AT); thus, a comparison of the accuracy and clinical efficacy of other fluoroscopy and anatomical landmark positioning methods was not conducted. Secondly, the sample size was small, and studies with larger samples are required in the future to compare the clinical effects of multiple fluoroscopy and anatomical landmark positioning methods. In addition, due to the limited sample size, we did not compare which quadrant exhibits a higher knee function score. Consequently, subsequent studies with a larger sample and a higher level of evidence may aid in understanding the optimal positioning. Lastly, the presence of observational bias is inevitable in this study.

## Conclusion

Clinical outcomes significantly improved and were similar in both groups. Nevertheless, the palpation of femoral anatomical landmarks exhibited superior convenience and efficiency for experienced sports medicine practitioners, and additionally, it frequently achieved a more isometric femoral footprint than fluoroscopic positioning in certain scenarios.

## Data Availability

All data supporting the findings of this study are contained within the article.

## Abbreviations

MPFL: medial patellofemoral ligament
IKDC: International Knee Documentation Committee
CT: computed tomography
3D-CT: Three-dimensional computed tomography
TT-TG: tibial tuberosity-trochlear groove
FME: femoral medial epicondyle
AT: adductor tubercle
M:MPFLR; M + T:MPFLR + TTO MPFLR: Medial patellofemoral ligament reconstruction
TTO: Tibial tubercle osteotomy
